# The Late Embryogenesis Abundant Protein Family in Cassava (*Manihot esculenta* Crantz): Genome-Wide Characterization and Expression during Abiotic Stress

**DOI:** 10.3390/molecules23051196

**Published:** 2018-05-17

**Authors:** Chunlai Wu, Wei Hu, Yan Yan, Weiwei Tie, Zehong Ding, Jianchun Guo, Guangyuan He

**Affiliations:** 1 Key Laboratory of Biology and Genetic Resources of Tropical Crops, Institute of Tropical Bioscience and Biotechnology, Chinese Academy of Tropical Agricultural Sciences, Xueyuan Road 4, Haikou 571101, China; wuchunlai19900109@126.com (C.W.); yoyoyan7758@163.com (Y.Y.); tieweiwei@itbb.org.cn (W.T.); dingzehong@itbb.org.cn (Z.D.); 2 Genetic Engineering International Cooperation Base of Chinese Ministry of Science and Technology, Chinese National Center of Plant Gene Research (Wuhan) HUST Part, Key Laboratory of Molecular Biophysics of Chinese Ministry of Education, College of Life Science and Technology, Huazhong University of Science and Technology (HUST), Wuhan 430074, China

**Keywords:** abiotic stress, cassava, characterization, genome-wide analysis, late embryogenesis abundant (LEA) protein

## Abstract

Late embryogenesis abundant (LEA) proteins, as a highly diverse group of polypeptides, play an important role in plant adaptation to abiotic stress; however, LEAs from cassava have not been studied in cassava. In this study, 26 LEA members were genome-wide identified from cassava, which were clustered into seven subfamily according to evolutionary relationship, protein motif, and gene structure analyses. Chromosomal location and duplication event analyses suggested that 26 *MeLEAs* distributed in 10 chromosomes and 11 *MeLEA* paralogues were subjected to purifying selection. Transcriptomic analysis showed the expression profiles of *MeLEAs* in different tissues of stem, leaves, and storage roots of three accessions. Comparative transcriptomic analysis revealed that the function of *MeLEAs* in response to drought may be differentiated in different accessions. Compared with the wild subspecies W14, more *MeLEA* genes were activated in cultivated varieties Arg7 and SC124 after drought treatment. Several *MeLEA* genes showed induction under various stresses and related signaling treatments. Taken together, this study demonstrates the transcriptional control of *MeLEAs* in tissue development and the responses to abiotic stress in cassava and identifies candidate genes for improving crop resistance to abiotic stress.

## 1. Introduction

Late embryogenesis abundant (LEA) protein was first found to accumulate during the late embryogenesis in cotton seed [1]. Subsequently, they were identified from various plant species, including rice, *Arabidopsis*, maize, barley, wheat, sunflower bean, and *Brassica napus* [2,3,4,5,6,7,8]. Additionally, LEA proteins also exist in other species, such as nematodes and chironomids [9,10]. LEA proteins have small molecular weight and a high degree of hydrophilicity [11,12]. Based on the eight conserved Pfams (PF04927, PF00257, PF03760, PF03168, PF03242, PF02987, PF00477, PF10714), plant LEA proteins can be separated into eight families: seed maturation protein (SMP), dehydrin, LEA_1, LEA_2, LEA_3, LEA_4, LEA_5, and LEA_6 [13].

Most of the LEA protein families have a unique subcellular localization, which is involved in protecting the stability of cells under stress conditions [3,14]. Accumulated evidence has revealed the biochemical mechanisms underlying their function on protecting plants from dehydration. Firstly, LEA proteins function as a protectant of membranes and biomolecules [14]. Secondly, LEA proteins, as intrinsically-disordered proteins, protect enzymes from induced aggregation by space-filling of LEA proteins, thus decreasing the rate of collisions between aggregating proteins [15,16]. Thirdly, LEA proteins aid in sequestration of calcium and metal ions, a benefit for signaling transduction [17]. Lastly, LEA proteins play a role in the formation of the glassy state, contributing to sugar accumulation in the cytoplasm of plants [18].

Moreover, genetic evidences also support the function of LEA proteins on abiotic stress response, including dehydration, osmotic, drought, salt, and chilling. Overexpression of the *OsLEA3-2* was able to enhance salt and drought tolerance in transgenic rice plants [19]. *ZmLEA3* could increase the transgenic tobacco tolerance to low temperature, osmotic and oxidative stresses [20,21]. Overexpression of the *CuCOR19* in tobacco increased cold tolerance by inhibiting lipid peroxidation [22]. Overexpression of the wheat *WCOR410* improved freezing resistance in transgenic strawberry leaves [23]. *SiLEA14* conferred salt and osmotic tolerance in transgenic *Arabidopsis* and foxtail millet [24]. Overexpression of the *JcLEA* in transgenic *Arabidopsis* plants improved resistance to dehydration and salinity [25]. Transgenic Arabidopsis plants overexpressing the Group LEA_4 protein of *B. napus* enhanced salt and drought tolerance [26]. Together, these results suggested that LEA proteins play a positive role in plant response to abiotic stress.

To date, *LEA* genes have been genome-wide identified in various plant species, such as 51 *LEAs* in *Arabidopsis* [3], 39 *LEAs* in rice [2], 108 *LEAs* in *Brassica napus* [8], 23 *LEAs* in Moso Bamboo [27], 36 *LEAs* in *Brachypodium distachyon* [28], and 53 *LEAs* in *Populus trichocarpa* [29]. However, less is known about this family in the important tropical crop cassava. Cassava, the third most important crop after rice and maize in Asia, Africa, and Latin America, provides dietary carbohydrates for over 600 million people across the tropical and sub-tropical world [30,31]. Cassava has a tolerance to drought and low-fertility soils due to the efficient use of heat, light, and water resources [32]. Transcriptomic analysis indicated that genes involved in abscisic acid (ABA) and ethylene biosynthesis, protein degradation, lipid metabolism, and second metabolism of flavonoids were significantly induced after osmotic treatment in cassava [33]. Some genes related to carbohydrate metabolism, ABA and salicylic acid metabolism, and calcium signaling were commonly regulated when cassava responded to cold and drought stresses [34]. lncRNAs associated with hormone signal transduction, secondary metabolites biosynthesis, and sucrose metabolism showed transcriptional changes after cold or drought treatment in cassava [35]. In addition, gene families involved in ABA and calcium signal transduction were identified from the cassava genome. Some members were widely responsive to drought, osmotic, cold, salt, and ABA at transcriptional levels [36,37,38,39]. Together, these evidences suggested that phytohormone metabolism, signaling transduction and secondary metabolites biosynthesis play a crucial role in cassava tolerance to abiotic stress. Due to the significance of LEA proteins in plant adaptation to abiotic stress, the LEA family was selected for systematic analysis in cassava.

In this study, an effort was made to identify MeLEAs from the cassava genome and investigate their phylogenetic relationship, conserved motif, gene structure, chromosomal location, duplication event, and expression profiles in distinct tissues and in response to drought, salinity, osmotic, cold, ABA, and H_2_O_2_ treatments. This systematic study should increase our understanding of LEA proteins related to abiotic stress response and lay a foundation for genetic improvement of crops.

## 2. Results

### 2.1. Identification and Phylogenetic Analysis of MeLEAs

**Figure 1:**
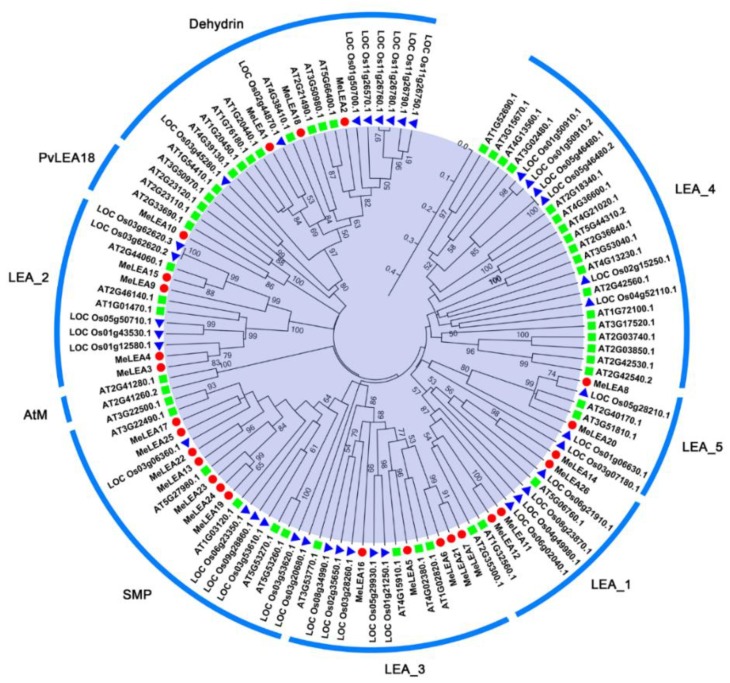
Evolutionary analysis of the LEAs from cassava, rice, and *Arabidopsis*. A total of 26 LEAs from cassava, 37 LEAs from rice and 51 LEAs from Arabidopsis were used to create the neighbour-joining (NJ) tree with 1000 bootstraps. Red circle, LEAs in cassava; Green square, LEAs in *Arabidopsis*; Blue triangle, LEAs in rice.

A total of 26 LEA proteins were identified from cassava based on HMM searches and conserved domain validation. The accession number and predicted characteristic of all the MeLEA proteins are shown in Supplementary Table S1. To investigate the phylogenetic relationship of LEA proteins, an unrooted neighbour-joining (NJ) tree was constructed with the complete LEA protein sequences from *Arabidopsis*, rice, and cassava (Figure 1). The LEA family were clustered into nine subgroups, including LEA_1, LEA_2, LEA_3, LEA_4, LEA_5, Dehydrin, PvLEA18, AtM, and SMP. The 26 MeLEAs were divided into seven subfamilies, including LEA_1, LEA_2, LEA_3, LEA_5, PvLEA18, Dehydrin and SMP. The SMP and LEA_3 families were the largest, with seven and five LEA members in cassava, respectively. By contrast, the PvLEA18 group had only one MeLEA member, MeLEA10.

### 2.2. Conserved Motifs and Gene Structure of MeLEAs

**Figure 2:**
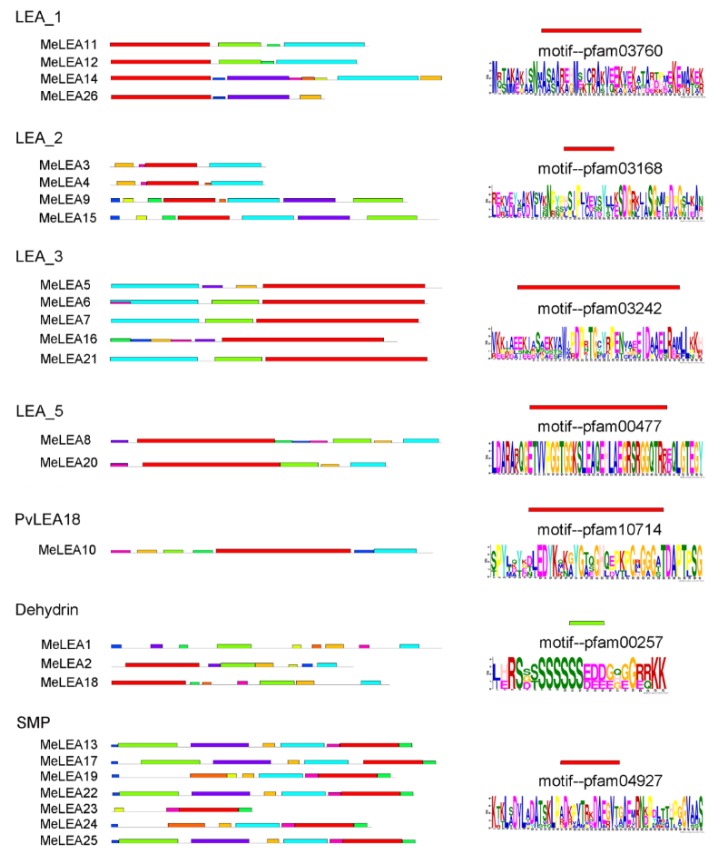
The conserved motifs of the MeLEAs according to the evolutionary classification. The conserved motifs were identified by the MEME database. The coloured boxes represent conserved motifs and grey lines represent the non-conserved sequences. The representative motif of each subfamily was annotated by PFAM databases with Pfam codes.

To understand the structural features of the MeLEAs, conserved motifs were identified according to phylogenetic relationship. Since the 26 MeLEAs did not share high similarity, each subfamily was respectively submitted to MEME database and a total of 10 conserved motifs of MeLEAs were identified (Figure 2). Subsequently, the typical motif for each subfamily was annotated with the Pfam database. The red motif that encoded a conserved LEA domain by Pfam annotation existed in six subfamilies (LEA_1, LEA_2, LEA_3, LEA_5, SMP and PvLEA18). The conserved LEA domain in the Dehydrin subfamily was represented by green motif. These results suggested that the identified MeLEAs have the typical motifs of LEA family [8]. Generally, the LEA proteins belonging to the same subfamily shared similar motif organization, further supporting the phylogenetic classification [2,8,27].

**Figure 3:**
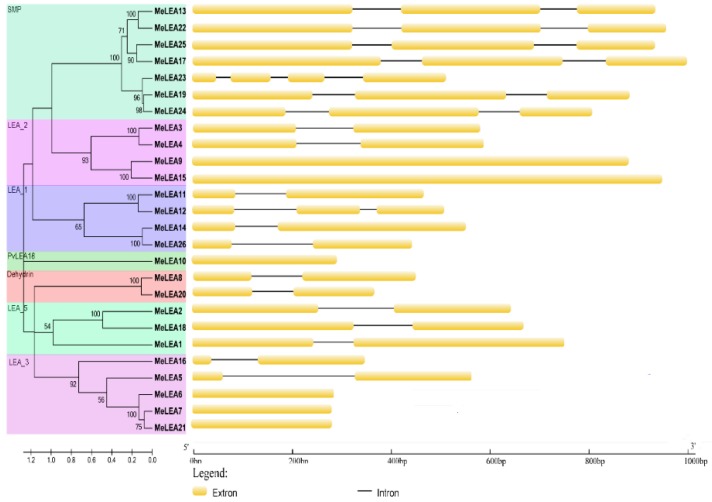
The exon-intron structure of the *MeLEAs* based on the evolutionary relationship. Exon-intron analysis of *MeLEAs* were performed with GSDS database. The back lines and the yellow boxes represent introns and exons, respectively.

Additionally, exon-intron organization of *MeLEAs* was analysed (Figure 3). The subgroup SMP was intron-rich with 2–3 introns, whereas other subgroups were intron-less with 0–1 intron, except for *MeLEA12* with two introns. Generally, *MeLEA* genes in the same subfamily has similar exon-intron feature, which evidences their close phylogenetic relationship and the classification of subgroups.

### 2.3. Chromosomal Distribution and Duplication Pattern of MeLEAs

**Figure 4:**
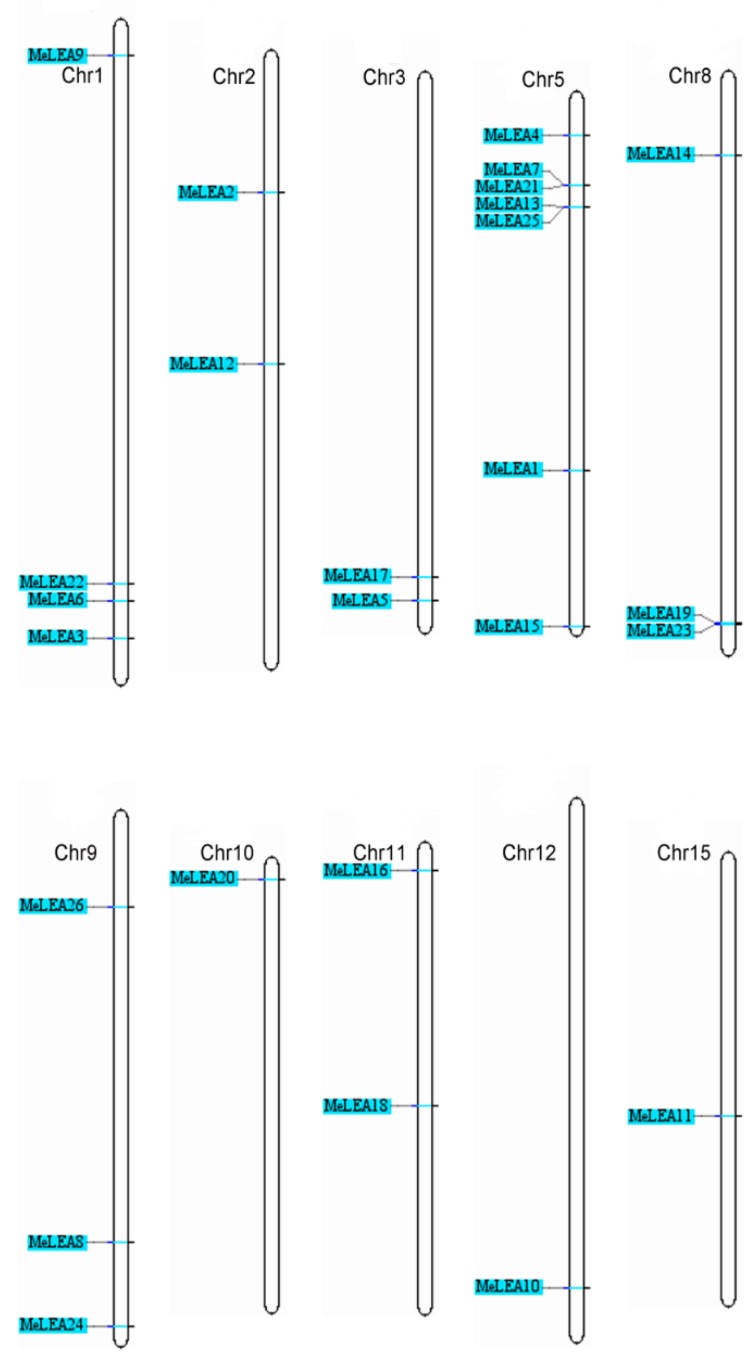
Distribution of the *MeLEAs* on chromosomes. The 26 *MeLEAs* were mapped to 10 chromosomes based on their chromosomal information of cassava.

To study the distribution of *MeLEAs*, the locations of the 26 *MeLEA* genes in chromosomes were identified (Figure 4). The *MeLEAs* were mapped to chr1, 2, 3, 5, 8, 9, 10, 11, 12, and 15. The PvLEA18 subfamily that contained only one gene *MeLEA10* was located on chromosome 12. LEA_2 and LEA_3 subfamilies appeared in the chromosome 1 and 5, except for *MeLEA5* and *MeLEA16*. The SMP subfamily which had seven members was distributed in chromosome 1, 3, 5, 8, and 9. LEA_1 subfamily contained four *MeLEAs* was placed in chromosome 2, 8, 9, and 15. The chromosomal location of LEA_5 subfamily were in chr9 and chr10. Dehydrin subfamily which contained *MeLEA1*, *2*, *18* was distributed in chromosome 5, 2, 11, respectively. 

**Figure 5:**
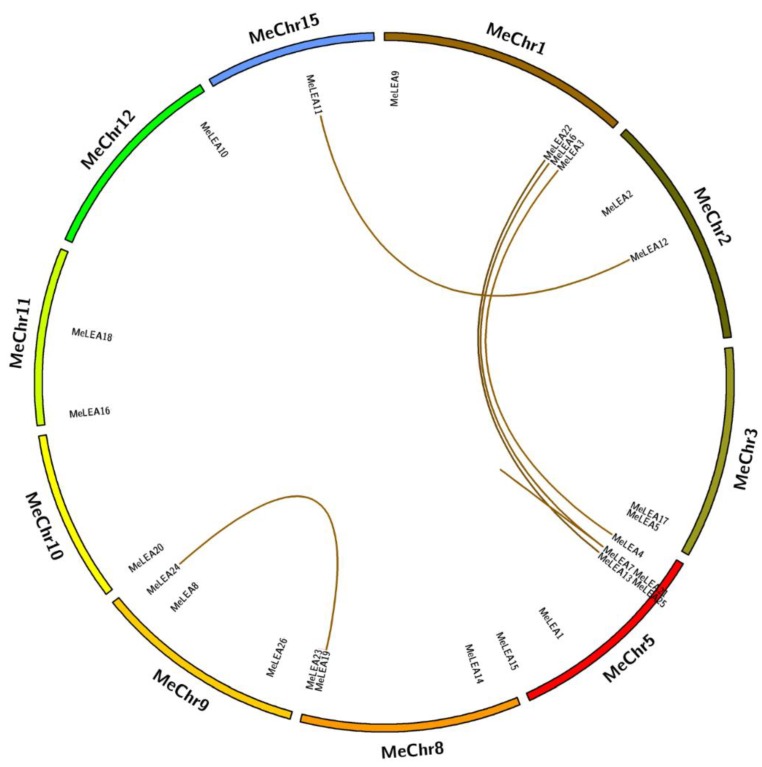
Segmental duplication of *LEA* genes in cassava. Chromosomes are illustrated in different colour in circular form using the program Circos. Segmental duplicated *MeLEAs* are connected by yellow lines.

To study the expansion of the *MeLEA* family in cassava, all nucleotide sequences of *MeLEAs* were aligned to identify their duplication patterns. Five duplication events involving 11 paralogues were identified (*MeLEA3/MeLEA4*, *MeLEA11/MeLEA12*, *MeLEA13/MeLEA22*, *MeLEA19/MeLEA24* and *MeLEA6/MeLEA7/MeLEA21*), which evidenced that segmental duplications had an important role in *MeLEAs* expansion in the cassava genome (Figure 5).

Further, non-synonymous (*Ka*) and synonymous (*Ks*) values were calculated for the duplicated *MeLEA* genes based on nucleotide sequences. The pairwise comparison data revealed that the *Ka/Ks* ratios in all the paralogous genes were between 0.2 and 0.45, showing the purifying selection of these genes (Supplementary Table S2).

### 2.4. Expression Profiles of LEA Genes in Different Cassava Tissues

**Figure 6:**
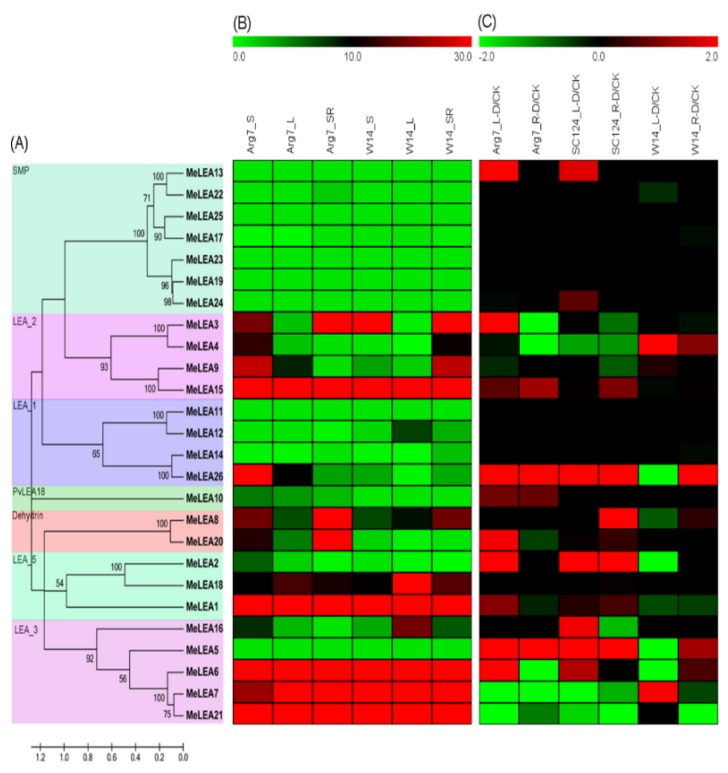
Expression patterns of *MeLEAs* in different tissues and in response to drought stress. (**A**) Evolutionary analysis of the LEAs from cassava; (**B**) Expression profiles of the *MeLEAs* in various tissues of Arg7 and W14. FPKM value is used to create the heat map with Mev 4.9.0 software. The color scale indicates the expression levels of *MeLEAs*. S, stem; L, leaf; SR, storage root. The FPKM value of the genes that was not covered in the transcriptomic datasets was set as “0”; and (**C**) expression profiles of the *MeLEAs* in leaves and roots of Arg7, SC124, and W14 after drought treatment. Log2-based fold changes was used to create the heat map with Mev 4.9.0 software. The FPKM value of the genes that were not covered in the transcriptomic datasets was set as “0”. Changes in gene expression are shown in color as the scale. L, leaf; R, root; D, drought treatment; CK, control check, showing leaf and root of cassava varieties under normal condition.

To examine the transcriptional levels of the *LEA* genes in diverse tissues of cassava, the storage roots, stems, and leaves of W14 and Arg7 were collected to perform RNA-seq analysis (Figure 6B; Supplementary Table S3). Nineteen out of the 26 *MeLEAs* had the corresponding transcripts data, while the remaining 7 *MeLEA* genes (*MeLEA-5*, *-11*, *-13*, *-19*, *-23*, *-24*, *-25*) were uncovered in the transcriptome dataset of different tissues of Arg7 and W14. These 7 *MeLEA* genes may have no, or low, expression levels in different tissues of cassava. In these 19 *MeLEA* genes, eight (42%), seven (37%), and five (26%) *MeLEAs* had high expression levels (FPKM value > 20) in storage roots, stems and leaves of Arg7, respectively. The *MeLEAs* with high expression levels (FPKM value > 20) in storage roots, stems and leaves of W14 were seven (37%), six (32%), and six (32%), respectively. Notably, *MeLEA1* in the LEA_5 subgroup, *MeLEA15* in the LEA_2 subgroup, and *MeLEA-6*, *-7*, *-21* in the LEA_3 subgroup showed high expression abundance (FPKM value > 20) in all the tested tissues of Arg7 and W14. These *LEA* genes may be beneficial for cassava tissue development and function. 

### 2.5. Expression Profiles of LEA Genes in Response to Drought Stress

To further investigate the possible role of *MeLEAs* under drought stress, water withholding was applied to a wild subspecies W14 and two cultivated varieties Arg7 and SC124. Then, total RNA was collected from roots and leaves for transcriptomic analyses (Figure 6C; Supplementary Table S4). According to the transcriptome data, 21 out of 26 *MeLEAs* had the corresponding expression data, while the rest of five *MeLEAs* (*MeLEA-11*, *-12*, *-19*, *-23*, *-25*) were not covered in the transcriptomic datasets. In W14, three (14%) and two (10%) *MeLEA* genes were transcriptionally up-regulated (log2-based fold change > 1) by drought stress in roots and leaves, respectively. In Arg7 variety, three (14%) and seven (33%) *MeLEA* genes were induced (log2-based fold change > 1) after drought stress in roots and leaves, respectively. In SC124 variety, four (19%) and six (29%) *MeLEA* genes showed up-regulation (log2-based fold change > 1) by drought stress in roots and leaves, respectively. Additionally, *MeLEA5* and *MeLEA26* were induced (log2 based fold change > 1) by drought in the two cultivated varieties. Together, these results revealed that the number of *LEA* genes induced by drought was more in Arg7 and SC124 than that in W14.

### 2.6. Expression Profiles of MeLEA Genes upon Exposure to Various Stresses and Related Signaling

**Figure 7:**
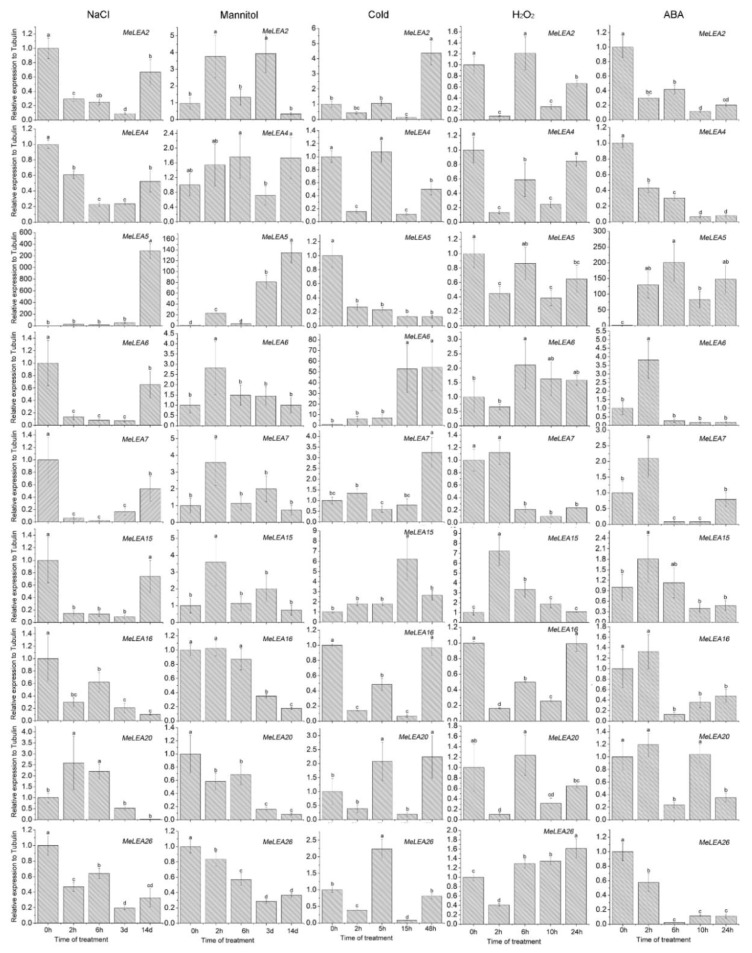
Expression profiles of the *MeLEAs* in leaves under salt, osmotic, cold, H_2_O_2_, and ABA treatments. NTC (no treatment control) was normalized as “1”. Data are means ± SD of *n* = 3 biological replicates. Means denoted by the same letter do not significantly differ at *p* < 0.05 as determined by Duncan’s multiple range test.

Based on the RNA-seq data, 9 genes (*MeLEA-2*, *-4*, *-5*, *-6*, *-7*, *-15*, *-16*, *-20*, *-26*) were induced by drought stress in different cassava accessions, which were chosen for further examination of their transcriptional patterns after salt, osmotic, cold, and H_2_O_2_ and ABA treatments (Figure 7). Under salt treatment, *MeLEA5* showed up-regulation at 14 d and *MeLEA20* showed up-regulation at 2 h and 6 h. In contrast, *MeLEA-2*, *-4*, *-5*, *-6*, *-7*, *-15*, *-16*, *-26* showed down-regulation at several treated time points. Under osmotic treatment, *MeLEA2* transcripts increased at 2 h and 3 d, *MeLEA5* at 2 h, 3 d, and 14 d, and *MeLEA-6*, *-7*, *-15* at 2 h. Conversely, the transcripts of *MeLEA-16*, *-20*, *-26* decreased at 3 d and 14 d. In response to cold stress, *MeLEA6* at 15 h and 48 h, *MeLEA7* at 48 h, *MeLEA15* at 15 h, and *MeLEA20* at 5 h and 48 h showed induction, whereas *MeLEA-4*, *-5*, *-16* exhibited repression. Under H_2_O_2_ treatment, *MeLEA6* at 15 h and *MeLEA15* at 2 h was induced, while *MeLEA-2*, *-4*, *-5*, *-7*, *-16*, *-20* were repressed at several treated time points. Treatment with ABA induced the expression of *MeLEA5* during 2 h–24 h, *MeLEA6* at 2 h, and *MeLEA15* at 2 h, but repressed the expression of *MeLEA-2*, *-4*, *-26* at all the treated time points, *MeLEA16* during 6 h–24 h, and *MeLEA20* at 6 h and 24 h. These results indicated that *MeLEAs* may be responsive to various stresses or signals, indicating their possible role in multiple signaling pathways in cassava.

## 3. Discussion

Due to the significant roles of LEA proteins in abiotic stress response and the typical feature of drought tolerance of cassava, it is requisite to systematically investigate the potential role of LEAs. In this study, we identified 26 MeLEAs from the cassava genome, which is shrunk in comparison to LEA members from *Arabidopsis* [3], rice [2], *Brassica napus* [8], *Brachypodium distachyon* [28], and *Populus trichocarpa* [29], but expanded compared with that from moso bamboo [27]. Previous phylogenetic analyses indicated that LEAs in most plant species contained eight subfamilies (LEA_1, LEA_2, LEA_3, LEA_4, LEA_5, PvLEA18, dehydrin, and SMP) [8]. MeLEAs were classified into seven subfamilies, among which the LEA_4 subfamily was not found in cassava (Figure 1). LEAs in *Arabidopsis* were classified into nine subfamilies (LEA_1, LEA_2, LEA_3, LEA_4, LEA_5, PvLEA18, dehydrin, SMP, and AtM), in which AtM was unique to the Brassicaceae [3]. Additionally, a total of 34 LEAs in rice were grouped into seven families (LEA_1, LEA_2, LEA_3, LEA_4, LEA_5, dehydrin, and SMP), whereas the PvLEA18 subfamily was lacking [2]. These evidences suggested that the subfamily variation of the LEA family existed in some plant species. Most of the *LEA* genes (25/26) harbored less introns (0–2) in cassava (Figure 3), which is in accord with the intron feature of *LEAs* in other plant species, such as 21/23 *PeLEAs*, 92/108 *BnLEAs*, and 39/39 *OsLEAs* with 0–2 introns [2,8,29]. All the *MeLEAs* in SMP subfamily showed intron-rich feature (containing 2–3 introns) and low/no expression in different tissues. In contrast, *MeLEA15* in LEA_2 subfamily and *MeLEA-6*, *-7*, *-21* in the LEA_3 subfamily did not contain intron, but had high expression levels in various tissues (Figure 3 and Figure 6). These results suggested that intron structure variation is associated with the expression patterns of *MeLEAs*. Additionally, MeLEAs belonging to the same subfamily had similar motifs and exon-intron compositions, further supporting the phylogenetic classification of MeLEAs (Figure 2 and Figure 3). 

The expansion of a gene family occurs via three modes: segmental duplication, tandem duplication, and whole-genome duplication [40,41]. It was necessary to analyze the duplication events of *LEA* genes in cassava. Firstly, the chromosomal distribution of the *LEAs* in cassava was investigated, which was widely distributed in the genome, and the similar result was observed in Arabidopsis, rice and *Brassica napus* (Figure 4) [2,3,8]. Subsequently, five pairs of paralogous genes (*MeLEA3/MeLEA4*, *MeLEA11/MeLEA12*, *MeLEA13/MeLEA22*, *MeLEA19/MeLEA24*, and *MeLEA6/MeLEA7/MeLEA21*) were identified from cassava, suggesting that segmental duplication may be the main expansion mechanism for *MeLEAs* (Figure 5). Further *Ka/Ks* ratio calculation showed the paralogues of *MeLEAs* were subjected to purifying selection (Supplementary Table S2). This is consistent with the *LEA* family expansion in *Brassica napus* and *B. distachyon* [8,28]. Of these paralogous gene pairs, *MeLEA3/MeLEA4* showed similar exon-intron organization (two exons), whereas distinct expression profiles in different tissues; *MeLEA11/MeLEA12* showed different exon-intron organization, but commonly low/no expression in different tissues; *MeLEA13/MeLEA22* and *MeLEA19/MeLEA24* commonly had three exons and low/no expression in different tissues; and *MeLEA6/MeLEA7/MeLEA21* commonly displayed 1 exons and high expression in different tissues. Additionally, these paralogous gene pairs exhibited different expression patterns after drought treatment in different cassava accessions (Figure 3 and Figure 6C). These results indicated that segmental duplication-driven expansion resulted in similar gene structure and expression diversity of most paralogous *MeLEAs*. This is in accord with the current opinion that gene duplication generates new genetic diversity as a basis for evolutionary innovation in eukaryotes [42].

Genome-wide expression analysis have demonstrated the significant response of *LEA* genes to drought or osmotic stress in various species, including *Arabidopsis*, rice, *Moso bamboo*, and sweet orange [2,3,27,43]. In this study, we found that some *MeLEA* genes, including *MeLEA-2*, *-5*, *-6*, *-13*, *-26*, were induced (Log_2_FPKM > 1) by drought in different cassava accessions. Additionally, we observed that the total number of *MeLEAs* up-regulated by drought was greater in Arg7 and SC124 than that in W14, indicating the comprehensive activation of *LEAs* in Arg7 and SC124 (Figure 6C). This indicated that *LEAs*-mediated drought responsive mechanism are differentiated between wild subspecies and cultivated varieties. Due to the differences for the genetic background between wild species (W14) and cultivated varieties (Arg7 and SC124) [44], the contribution of *MeLEA* genes to drought tolerance of cassava is elucidated.

Previous studies demonstrated that LEAs could widely participate in plants response to abiotic stress and hormones [2,3,8,19,20,21,22,23,24,25,26,27,28,29]. Here, we examined the expression of nine genes (*MeLEA-2*, *-4*, *-5*, *-6*, *-7*, *-15*, *-16*, *-20*, *-26*) under salt, osmotic, cold, and H_2_O_2_ and ABA treatments. The results showed that almost all the *MeLEA* genes could be induced at least by one of the treatment (Figure 7). Notably, *MeLEA5* was commonly inducted by ABA, salt, and osmotic treatments; *MeLEA6* was commonly up-regulated by ABA, cold, and osmotic treatments; *MeLEA7* and *MeLEA15* were commonly up-regulated by cold and osmotic treatments; and *MeLEA20* was commonly induced by cold and salt treatments. In rice, *OsLEA-5*, *-12*, *-29* showed up-regulation after osmotic, salt, and ABA treatments [2]. In *Moso bamboo*, 10 genes showed induction after both dehydration and cold stresses [19]. These evidences supports that a single *MeLEAs* is involved in various stresses or signals and *MeLEA-5*, *-6*, *-7*, *-15*, *20* can serve as candidates for genetic improvement of crop tolerance to multiple stresses. 

## 4. Materials and Methods 

### 4.1. Plant Materials and Treatments

The characteristics of W14, SC124, and Arg7 cassava accessions were described in previous studies [45,46]. W14 (*M. esculenta* ssp. *flabellifolia*) is a wild subspecies of cultivated cassava. Arg7 is a variety containing elite agronomic traits, including a certain level of growth under moderate drought stress. SC124 is a widely planted cassava cultivar in China and can survive in prolonged severe drought stress. All the cassava plants were cultured with soil and vermiculite (1:1) under the glass house in the CATAS (Chinese Academy of Tropical Agricultural Sciences, Haikou, China). To analyze transcripts of *MeLEAs* in distinct tissues at the early development stage, stems (90 days old), leaves (90 days old), and storage roots (150 days old) were gathered from Arg7 (cultivated variety) and W14 (wild subspecies) under normal conditions. To examine the transcriptional levels of *MeLEAs* under drought stress, cassava plants (90 days old) of W14, Arg7, and SC124 were subjected to water withholding for 12 days, and the leaves and roots were collected for RNA sequencing. To detect the expression levels of *MeLEAs* under abiotic stress, leaves were collected from Arg7 (60 days old) for quantitative real-time PCR (qRT-PCR) detection. For salt and osmotic treatments, cassava seedlings grew in soil irrigated with 300 mM NaCl or 200 mM mannitol for 14 days. For cold treatment, cassava seedlings were cultured in an incubator with low temperature (4 °C) for 48 h. For ABA and H_2_O_2_ treatments, cassava seedlings grew in soil irrigated with 100 μM ABA or 10% H_2_O_2_ for 24 h.

### 4.2. Identification and Phylogenetic Analyses

The whole LEA protein sequences in *Arabidopsis* and rice were obtained from the *Arabidopsis* information resource (TAIR) and rice genome annotation project (RGAP) databases, respectively [47,48]. The whole genome and protein sequences of cassava were downloaded from the Phytozome 12.0 cassava database (Lawrence Berkeley National Laboratory, Berkeley, CA, USA). MeLEAs were identified by hidden Markov model (HMM) profiles built from the known LEA protein sequences [49]. To confirm the LEAs of cassava, the candidate MeLEAs were further analyzed using the conserved domain search (CDD), simple modular architecture research tool (SMART), and the basic local alignment search tool (BLAST) databases [50,51,52]. The unrooted phylogenetic tree was created with LEA protein sequences from cassava, *Arabidopsis*, and rice by ClustalX 2.0 (Tokyo Metropolitan University, Tokyo, Japan) and MEGA 6.0 software packages (University College Dublin, Dublin, Ireland) bootstrap values for 1000 replicates) [53,54]. 

### 4.3. Protein Properties and Gene Structure Analysis

The ExPASy database (Swiss Institute of Bioinformatics, Lausanne, Switzerland) and DNASTAR software packages (DNASTAR, Madison, WI, USA) were used to predict the molecular weight and isoelectric points of MeLEA proteins [55,56]. The MEME database was employed to identify the conserved motifs of MeLEA proteins [57]. Then, all the structural motifs were annotated by SMART, PFAM, and InterProScan databases [13,49,58]. The exon/intron organization of *MeLEAs* were identified by the Gene Structure Display Server (GSDS) database [59].

### 4.4. Chromosomal Location and Duplication Pattern Analysis

The chromosomal location of *MeLEA* genes was identified according to the data information obtained from the Phytozome 12.0 cassava database. Paralogous *LEA* genes were selected according to calculation of sequence identity and the phylogenetic relationship. Gene duplication events of paralogous *LEA* genes in cassava were identified based on the following two rules: (1) the alignment covered >80% of the longer gene; (2) the aligned region had an identity >80% [60,61]. The *Ks* (synonymous substitution) and *Ka* (non-synonymous substitution) were calculated by DnaSP 5.0 software (Universitat de Barcelona, Barcelona, Spain) [62]. A *Ka/Ks* ratio < 1 indicates a purifying selection, a *Ka/Ks* ratio = 1 indicates a neutral selection, and a *Ka/Ks* ratio > 1 indicates a positive selection [63,64].

### 4.5. Transcriptomic Analysis

Samples were gathered to extract total RNA with plant RNA extraction kit (TIANGEN, Beijing, China). The total RNA of each sample was used to construct cDNA libraries. Then, Illumina GAII platform (Illumina, San Diego, CA, USA) was used to perform high throughput sequencing according to manufacturer’s RNA-seq protocol. Adapter sequences were cleaned by FASTX-toolkit in the raw reads [65]. To obtain high quality sequences, FastQC was used to remove low quality sequences [66]. Then, clean reads were mapped on the cassava genome by Tophat v.2.0.10 (Johns Hopkins University, Baltimore, MD, USA) [67]. Cufflinks was used to performed transcriptome assemblies [68]. Finally, FPKM (fragments per kilobase of transcript per million mapped fragments) values were calculated to create heat map with MeV 4.9 software (CCCB, Boston, MA, USA). 

### 4.6. qRT-PCR Analysis

Expression of *MeLEAs* in response to various stimuli were determined by quantitative real-time PCR (qRT-PCR) on Stratagene Mx3000P instrument (Stratagene, Santiago, CA, USA). AlleleID6.0 was used to design gene-specific primer pairs that were further validated by melting curve and agarose gel electrophoresis (Supplementary Table S5). Each reaction system had a volume of 10 μL containing 0.5 μL of cDNA, 1 μL of gene-specific primers, 3.5 μL of RNA-Free water and 5 μL of SYBR^®^ Premix Ex Taq™ (TaKaRa, Tokyo, Japan). The PCR conditions were implemented as follows: 5 min at 95 °C; 40 cycles of 10 s at 95 °C, 15 s at 55 °C and 20 s at 72 °C; 60 s at 95 °C, 30 s at 55 °C and 30 s at 95 °C. The Tubulin gene was used to normalize as a reference. The 2^−ΔΔ*C*t^ approach was carried out to calculate the relative expression of *MeLEA* genes. The relative expression levels of *MeLEA* genes in each time point was calculated according to the control and treated samples. Each sample has three replicates and three biological experiments were performed.

## 5. Conclusions

In conclusion, this study identified 26 LEAs from cassava and studied their classification by phylogenetic, protein motif, and gene structure analyses. Chromosomal location and duplication event analysis revealed that 26 *MeLEAs* were distributed in 10 chromosomes and 11 *LEAs* were paralogues which were subjected to purifying selection. Transcriptomic analyses revealed the tissue expression and drought response diversity of *MeLEAs* in wild species and cultivated varieties. Several *MeLEA* genes were observed to respond to multiple abiotic stresses, and H_2_O_2_ and ABA signaling. This systematic research will provide a solid foundation for understanding the function of *LEA* genes and *LEAs*-mediated abiotic stress response in cassava.

## Supplementary Materials

The following are available online. Table S1. Characteristics of *LEAs* in cassava; Table S2. The *Ka*/*Ks* ratios of duplicated *LEA* genes in cassava; Table S3. The expression profiles of the cassava *LEA* genes in different tissues; Table S4. The expression profiles (log2-based fold changes) of the cassava *LEA* genes after drought treatment; Table S5. Primers used in qRT-PCR analysis.
